# *C. elegans* epicuticlins define specific compartments in the apical extracellular matrix and function in wound repair

**DOI:** 10.1242/dev.204330

**Published:** 2024-10-23

**Authors:** Murugesan Pooranachithra, Erin M. Jyo, Nicolas Brouilly, Nathalie Pujol, Andreas M. Ernst, Andrew D. Chisholm

**Affiliations:** ^1^ Department of Cell and Developmental Biology, School of Biological Sciences, University of California San Diego, La Jolla, CA 92093, USA; ^2^ Aix Marseille University, CNRS, IBDM, 13009, Marseille, France; ^3^ Aix-Marseille Université, INSERM, CNRS, CIML, Turing Centre for Living Systems, 13009, Marseille, France

**Keywords:** Tandem repeat proteins, Disordered proteins, Permeability barrier, Epithelia, Lipid layers, Dauer larva

## Abstract

The apical extracellular matrix (aECM) of external epithelia often contains lipid-rich outer layers that contribute to permeability barrier function. The external aECM of nematodes is known as the cuticle and contains an external lipid-rich layer – the epicuticle. Epicuticlins are a family of tandem repeat cuticle proteins of unknown function. Here, we analyze the localization and function of the three *C. elegans* epicuticlins (EPIC proteins). EPIC-1 and EPIC-2 localize to the surface of the cuticle near the outer lipid layer, as well as to interfacial cuticles and adult-specific struts. EPIC-3 is expressed in dauer larvae and localizes to interfacial aECM in the buccal cavity. Skin wounding in the adult induces *epic-3* expression, and EPIC proteins localize to wound sites. Null mutants lacking EPIC proteins are viable with reduced permeability barrier function and normal epicuticle lipid mobility. Loss of function in EPIC genes modifies the skin blistering phenotypes of Bli mutants and reduces survival after skin wounding. Our results suggest EPIC proteins define specific cortical compartments of the aECM and promote wound repair.

## Teaser

**Summary:** Analysis of localization and function of epicuticlins, which are tandem repeat proteins associated with the lipid-rich epicuticle layer of the *C. elegans* cuticle.

## INTRODUCTION

Animal barrier epithelia contain a complex apical extracellular matrix (aECM) that provides structural integrity and forms part of the permeability barrier. Extracellular lipid layers are key components of the matrix permeability barrier; for example, the lamellar lipids and cornified lipid envelope of the mammalian stratum corneum (Jonca and Simon, 2023), the tear film lipid layer of the cornea (Pflugfelder and Stern, 2020), or the surfactant lipid layers of alveolar lung cells (Olmeda et al., 2017). The outer lipid layer of arthropods is known as the envelope (Locke, 2001), whereas the equivalent structure in nematodes is termed the epicuticle (Lee and Atkinson, 1976; Bird and Bird, 1991). Extracellular lipid layers can be generated by secretion of flattened lipid disks from organelles such as lamellar bodies (Menon et al., 2018) or from exosome-like vesicles (Tsarouhas et al., 2023). In the lung alveolar epithelium, lipid-binding proteins, such as saposins, play key roles in biogenesis of lipid disks (Sever et al., 2021). However, the composition and biogenesis of extracellular lipid layers and their interaction with other aECM compartments in general remain poorly understood.

We are interested in the *C. elegans* epicuticle as a model extracellular lipid layer that forms an outer subcompartment of the animal's aECM (reviewed by Sundaram and Pujol, 2024). Like other nematodes, *C. elegans* generates an epicuticle with trilaminar appearance in TEM ∼10-30 nm thick and evenly covering the rest of the body cuticle (Altun and Hall, 2008). More complex multilaminate or folded epicuticles have been seen in some parasitic species or stages (Sayers et al., 1984; Franz et al., 1987). In other nematodes, the epicuticle and its lipids have been implicated in host-pathogen interactions (Brivio and Mastore, 2020) and in resistance to abiotic stress (Bird and Buttrose, 1974; Wharton and Lemmon, 1998). Based on its trilaminar appearance in TEM and freeze-fracture EM (Peixoto and De Souza, 1995), a prevailing model is that epicuticle resembles a lipid bilayer with protein components.

Imaging, biochemical and genetic studies confirm that the epicuticle contains lipids. The *C. elegans* epicuticle can be stained by lipophilic dyes (Schultz and Gumienny, 2012), as in other nematodes (Kennedy et al., 1987). Lipophilic dye uptake by the epicuticle is selective and dye mobility is generally low, albeit varying with developmental stage (Proudfoot et al., 1993). Biochemical studies indicate the *C. elegans* epicuticle contains a complex mixture of polar lipids, phosphoglycerides, ceramides, sphingomyelin and cardiolipin (Blaxter, 1993; Bada Juarez et al., 2019). *C. elegans* lipid biosynthesis mutants display cuticle permeability defects, consistent with epicuticle lipids being required for permeability barrier function (Watts et al., 2003; Kage-Nakadai et al., 2010; Loer et al., 2015; Njume et al., 2022). It remains unclear how the epicuticle assembles or how it is attached to the rest of the cuticle.

In biochemical studies of the *C. elegans* cuticle, the epicuticle and underlying outer cortical layer form part of the BME-insoluble and collagenase-resistant fraction (Cox et al., 1981a), suggesting the epicuticle and associated proteins are not collagens. The insoluble fraction has a biased amino acid composition both in *C. elegans* (Cox et al., 1981a) and *Ascaris* (Fujimoto and Kanaya, 1973). Components of the insoluble fraction include zona pellucida (ZP) family cuticlins or cuticlin-like (CUT, CUTL) proteins (Sebastiano et al., 1991; Lassandro et al., 1994; Ristoratore et al., 1994). Monoclonal antibodies raised against the insoluble fraction of *Ascaris* cuticle identified a previously unreported protein AsCut, later renamed epicuticlin 1, localized to the epicuticle layer in *Ascaris* and *Brugia* (Bisoffi et al., 1996). *Ascaris* epicuticlin 1 is made up of seven near-perfect Ala- and Gly-rich tandem repeats of 49-51 amino acids, predicted to be intrinsically disordered and containing motifs (YGDE and GYR) found in some insect cuticular proteins (Cornman, 2010).

Epicuticlin-related tandem repeat proteins are widespread in nematodes (Betschart et al., 2022) but their *in vivo* functions have not been analyzed. Here, we have analyzed the three *C. elegans* epicuticlin (EPIC) proteins. We show that *C. elegans* epicuticlins are localized to specific aECM compartments, consistent with localization to epicuticle or to the external cortical layer. Mutants lacking all three epicuticlins are viable with largely normal morphology and barrier function. TEM analysis indicates the epicuticle is present in *epic* triple-null mutants. EPIC-3 expression is confined to dauer larvae and induced by skin wounding in adults. Moreover, loss of EPIC protein function impairs survival after wounding. Our results suggest that the epicuticlins are not essential for epicuticle biogenesis but may act in specific epicuticle regions or in barrier repair.

## RESULTS

### Ultrastructural morphology of the *C. elegans* epicuticle

**Fig. 1.**
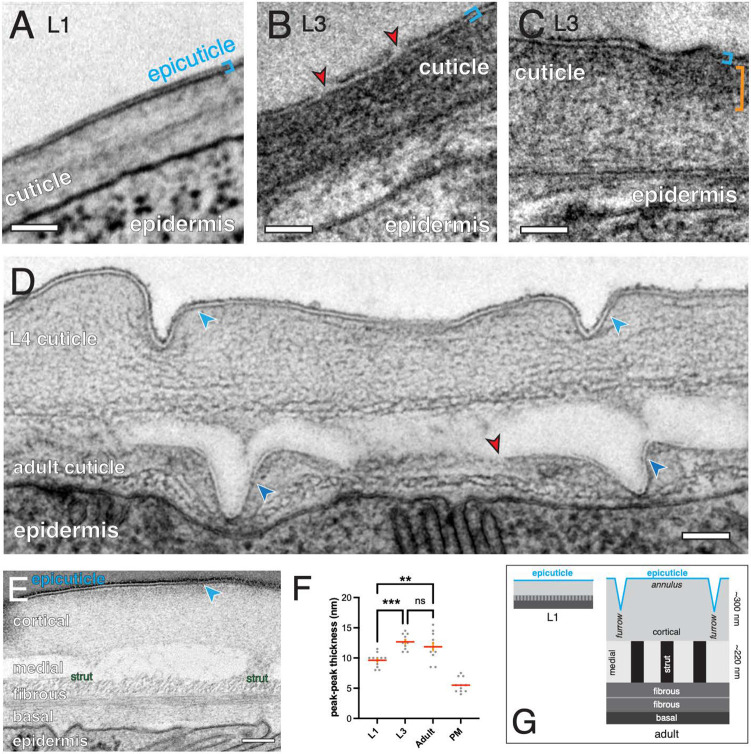
**Ultrastructure of the *C. elegans* epicuticle.** (A) Transverse section showing L1 epicuticle (blue brackets). Image from series S.E.M._L1_4, z62, described in Witvliet et al. (2021). Scale bar: 100 nm. L1 cuticle is ∼150 nm thick. Images in A-C are from midbody regions of cuticle over dorsal or ventral muscle quadrants. (B) Transverse section showing L3 epicuticle, blue brackets. Image from series TEM_L3, z140. Scale bar: 100 nm. Red arrowheads indicate discontinuities in the outer osmiophilic layer. (C) Example of differential staining of outer cortical cuticle (orange bracket). Image captured with a TEM_L3, z196. Scale bar: 100 nm. (D) Late L4 stage (∼L4.7) animal, midbody region, showing nascent adult cuticle under detached L4 cuticle. Image is a longitudinal section across one L4 annulus and two furrows. Blue arrowheads indicate epicuticle in L4 cuticle at furrows and on sides of furrows in nascent adult cuticle; red arrowhead indicates discontinuity where adult epicuticle may have not yet formed. Scale bar: 100 nm. (E) Longitudinal section of adult cuticle showing epicuticle, cortical layer, medial layer with two struts, fibrous layers and basal layer. Scale bar: 100 nm. Blue arrowhead indicates epicuticle. (F) Quantitation of epicuticle thickness in electron microscopy data, defined as peak-to-peak distance in line scans across the outer osmiophilic layers. ANOVA and Holm-Šídák post-test were used to test for significance. ns, not significant; **P<0.01; ***P<0.001. PM, plasma membrane bilayer thickness as measured using the same method in the same sections. Data are mean±s.e.m. (G) Schematic of cuticle layers in L1 versus adult. L1 cuticle is ∼150 nm thick, versus 1 μm in young adults. Epicuticle (blue) is not to scale. Adult schematic is longitudinal section.

The *C. elegans* epicuticle has been observed in ultrastructural studies (Cox et al., 1981b; Costa et al., 1997); however, details of its morphology have not been described at high resolution. We examined the epicuticle in TEM or SEM of wild-type adults and larval stages, using high-pressure freeze fixation (HPF) with OsO_4_ to highlight lipid membranes (see Materials and Methods). The trilaminate epicuticle forms two osmiophilic layers separated by an electron-lucent layer, in body cuticle regions of all stages examined (Fig. 1A-E). In some specimens, the outer osmiophilic layer was less stained than the inner layer; in other samples, the outer layer displayed gaps or discontinuities (e.g. Fig. 1B). Cortical cuticle staining was often non-uniform, with the outer cortical layer underlying the epicuticle being more electron-dense than the inner cortical layer (orange brackets, Fig. 1C).

In late L4 stages, the adult cuticle is synthesized underneath the L4 cuticle. In TEM of late L4 the adult epicuticle is most clearly seen at the sides of nascent furrows (Fig. 1D), suggesting epicuticle biogenesis may begin at furrows and later extend over annuli, reminiscent of the embryonic epicuticle (then termed the external cortical layer) (Costa et al., 1997). These observations also suggest that epicuticle forms early in cuticle biogenesis.

We measured epicuticle thickness as the distance between peak electron densities (i.e. midpoints of darkest regions) of the two osmiophilic layers, after classical methods for estimating plasma membrane thickness (Yamamoto, 1963). In L1 animals, the epicuticle was 9.6±0.3 nm thick (Fig. 1A; mean±s.e.m., *n*=12), whereas epicuticle of later larval stages or adults were slightly thicker (10-12 nm, *n*=11-14 per stage; Fig. 1E,F). Plasma membrane thickness in the epidermis in these sections was 5.5±0.3 nm, indicating the epicuticle is approximately twice as thick as a typical plasma membrane and ∼1% of overall adult cuticle thickness (Fig. 1G, not to scale).

### *C. elegans* EPIC proteins localize to specific compartments of the aECM

*C. elegans* encodes three EPIC proteins (epicuticlins) (Betschart et al., 2022) that, like *Ascaris* epicuticlin 1, are composed of perfect or near-perfect tandem repeats and are predicted to be disordered. *epic-1* and *epic-2* transcripts oscillate in larval development, with peak phase angles of ∼140°, approximately during ecdysis (Meeuse et al., 2020, 2023); *epic-3* transcripts are restricted to dauer or predauer larvae (Contrino et al., 2012). *epic-1* and *epic-2* transcription has been detected in the epidermis (Katsanos et al., 2021) and in adult interfacial epidermis (Ghaddar et al., 2023). These observations suggest that EPIC genes are expressed in multiple epidermal cell types.

**Fig. 2.**
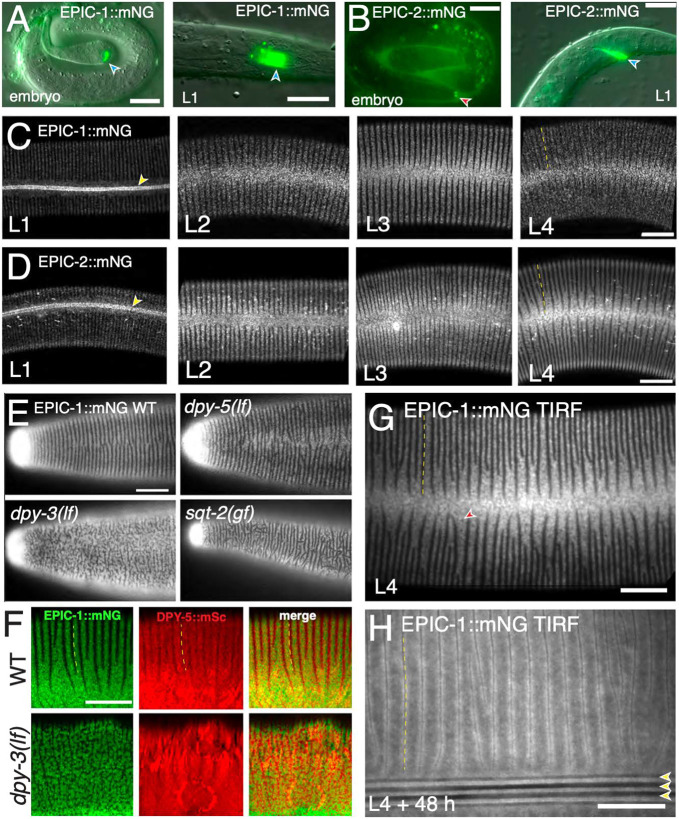
**Localization of EPIC-1 and EPIC-2 knock-ins in embryonic, larval and adult aECM.** (A) EPIC-1::mNG expression patterns in embryos and L1s. Images are wide-field DIC and fluorescence. EPIC-1::mNG was seen in rectal cuticle (blue arrowheads) and faintly in body cuticle. Right, EPIC-1::mNG in L1 larva. EPIC-1::mNG was most intense in distal rectal cuticle and, in two bright dots, possibly in rectal epithelial cells. Scale bars: 10 µm. (B) EPIC-2::mNG was detected in nose cuticle (red arrowhead) and faintly in the extra-embryonic space of late embryos (left). In early L1 animals (right), EPIC-2::mNG expression was most intense in rectal cuticle (blue arrowhead) and faintly in body cuticle. Scale bar: 10 µm. (C) EPIC-1::mNG in lateral cuticle of L1, L2, L3 and L4 stages, showing localization to L1 alae (yellow arrowhead) and granular localization in cortical annuli. Furrows in L4 are indicated by a dashed yellow line. Images were captured using Airyscan. Scale bar: 10 µm. Anterior is to the left and dorsal is upwards. (D) EPIC-2::mNG in lateral cuticle of larvae, showing localization to L1 alae and granular localization in annuli. Furrows are indicated by a dashed yellow line. (E) EPIC-1::mNG localization in L4 stage anterior cuticle in wild type and mutants *dpy-5(e61)*, *dpy-3(e182)* and *sqt-3(sc3)*. Images are confocal projections. Scale bar: 10 µm. (F) EPIC-1::mNG and DPY-5::mSc imaging in wild-type and *dpy-3(e182)* L4 stage; lateral views. Images were captured using Airyscan and are confocal single focal planes. Scale bar: 10 µm. EPIC-1::mNG-labeled annuli were in register with the DPY-5::mSc-enriched annuli; additional DPY-5 signal is underneath furrows; dashed lines indicate the furrows. (G,H) EPIC-1::mNG in TIRF microscopy of L4 and adults (48 h post L4). Red arrowhead indicates the hole in EPIC-1::mNG distribution in L4; furrows are indicated by dashed yellow lines. In adults (H), EPIC-1::mNG localized to the sides of the three alae ridges (yellow arrowheads; see also Fig. 5H). Images were captured using ring TIRF, surface plane with 100-nm penetration depth. Scale bar: 10 µm.

We tagged the EPIC proteins by CRISPR/Cas9 mediated insertion of mNeonGreen (mNG) at their C termini (see Materials and Methods). EPIC-1::mNG and EPIC-2::mNG were visible in late embryos (Fig. 2A) through to adult stage. In early L1 larvae, EPIC-1::mNG and EPIC-2::mNG were visible in the nose tip and rectal cuticle (Fig. 2A,B). From mid-L1 stage onwards, EPIC-1::mNG expression was seen in the body cuticle, including L1 alae, consistent with transcriptional reporters (Meeuse et al., 2020). In larval body cuticles, EPIC-1::mNG and EPIC-2::mNG were prominent in annuli and excluded from furrows (Fig. 2C,D). Within annuli, EPIC-1::mNG and EPIC-2::mNG had a granular appearance. Under similar imaging conditions, the cuticle displayed minimal autofluorescence in N2 controls (Fig. S1A). In adults, EPIC-1::mNG and EPIC-2::mNG diffusely localized to annuli and alae, as well as to struts (see below). We next examined EPIC-1::mNG in mutants affecting annulus (*dpy-5*) or furrow morphology (*dpy-3* and *sqt-2*) (Sandhu et al., 2021). In *dpy-5* mutants, the L4 localization of EPIC-1::mNG was largely normal (Fig. 2E). In contrast, *dpy-3* mutants displayed highly branched ‘labyrinthine’ patterns of EPIC-1::mNG; milder branching was seen in *sqt-2* mutants. We analyzed EPIC-1::mNG colocalization with an mScarlet (mSc) knock-in to the collagen DPY-5, which localizes to the fibrous layer beneath annuli, similar to DPY-13 (McMahon et al., 2003; Adams et al., 2023). In *dpy-3* mutants, the labyrinthine EPIC-1::mNG pattern resembled the cuticle surface of ‘furrow’ collagen mutants in TEM (Sandhu et al., 2021) or AFM (Aggad et al., 2023). This was distinct from DPY-5::mSc, which formed large amorphous annuli (Fig. 2F) consistent with previous observations of other annuli markers (Thein et al., 2003; Sandhu et al., 2021). These observations suggest that EPIC-1 localizes to a cortical aECM compartment.

To detect EPIC proteins at the cuticle surface, we used total internal reflection (TIRF) microscopy at maximum stringency and were able to visualize diffusely localized EPIC-1::mNG both at L4 stage and in adults (Fig. 2G,H; Fig. S1B-D). In variable-angle TIRF, diffuse EPIC-1::mNG was detected in the outer 100 nm of the cuticle in L4 and adults; in adults, punctate EPIC-1::mNG corresponding to struts (see below) was detected at 100-300 nm penetration depth (Fig. S1B,C). EPIC-2::mNG was also detected in adults under stringent TIRF conditions (Fig. S1D); autofluorescence was minimal in controls imaged under the same TIRF conditions (Fig. S1E). These observations indicate that diffuse EPIC-1::mNG localizes within 100 nm of the cortical surface.

**Fig. 3.**
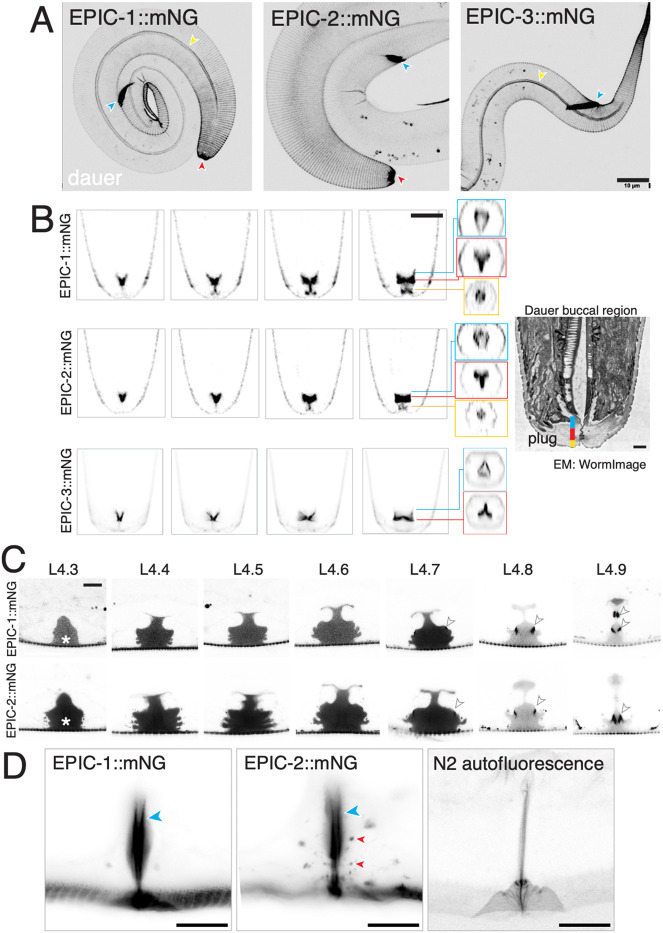
**EPIC::mNG knock-in localization in dauer larvae and hermaphrodite vulva.** (A) Images of dauer larvae bodies and head region, showing buccal area (red arrowhead) and rectal cuticle (blue arrowheads). EPIC proteins also localized to dauer alae (yellow arrowheads). Scale bar: 10 µm. (B) EPIC-1::mNG, EPIC-2::mNG and EPIC-3::mNG in the buccal region of dauer larvae. Anterior is down. Images are single planes of Airyscan *z* stacks, from lateral to medial. Scale bar: 5 μm. EPIC-1::mNG and EPIC-2::mNG localized to three regions, illustrated by three orthogonal sections labeled in yellow (∼0-0.5 μm from anterior nose tip), red (0.6-1.3 μm from tip) and blue (1.4-2.3 μm from tip). EPIC-3::mNG localized to the posterior two regions. The TEM image of the buccal plug is from WormImage N2_dauer_50-7-2_34 (www.wormimage.org). Scale bar: 1 µm, assuming an annulus width of 1 µm. Three subregions of the buccal plug are colored, with dimensions and symmetry corresponding to the regions of EPIC protein localization. (C) EPIC-1::mNG and EPIC-2::mNG in L4 vulva at different morphological substages, as defined by Mok et al. (2015). Both proteins were diffuse in the vulval lumen (asterisks) from L4.3 onwards and near the surface of vulC cells by L4.7 (arrowheads). EPIC-1::mNG also localized deeper in the vulva at L4.9 (top arrowhead). Images are single focal planes, captured using Airyscan. Scale bar: 5 μm. (D) EPIC-1::mNG and EPIC-2::mNG in adult vulva. Both knock-in proteins localized to the adult vulval cuticle (blue arrowheads). EPIC-2::mNG localized to putative secretory vesicles in vulval epithelium (red arrowheads). Images are lateral views of projections of five deep focal planes, captured using Airyscan. N2 imaged under the same conditions (right) shows a faint vulval cuticle autofluorescence. Scale bars: 10 µm.

In dauer larvae, EPIC-1::mNG, EPIC-2::mNG and EPIC-3::mNG each were highly expressed in the mouth and rectal cuticle, as well as in dauer alae and annuli (Fig. 3A). Dauer larvae contain a specialized thickened buccal plug that occludes the mouth (Albert and Riddle, 1983) (Fig. 3B). EPIC-1::mNG and EPIC-2::mNG localized in the plug and could be resolved into anterior thin filaments, a medial triradiate sheet (Y-shaped in cross section) and a posterior threefold symmetric sheet (Fig. 3B). The dimensions of the medial region of EPIC-1::mNG and EPIC-2::mNG are consistent with the thickened buccal plug cuticle. EPIC-3::mNG localization resembled that of the medial and posterior regions of EPIC-1::mNG and EPIC-2::mNG (Fig. 3B).

As well as being localized to the main body cuticle generated by hyp7 and seam cells, EPIC-1::mNG and EPIC-2::mNG were strongly expressed in cuticle generated by interfacial epidermal cells (e.g. the nose tip, rectum and vulva). In L4 substage 4.3-4.9 animals (Mok et al., 2015), EPIC-1::mNG and EPIC-2::mNG localized diffusely in the vulval lumen, as well as on the luminal surface near vulC cells (arrowheads, Fig. 3C). In adults, EPIC-1::mNG and EPIC-2::mNG localized to the apical surface of the vulval cuticle; EPIC-2 was also observed in punctate or filamentous structures (Fig. 3D). Taken together, EPIC proteins localize to a distinctive set of aECM compartments in multiple stages and regions. To understand the biochemical properties of EPIC::mNG proteins, we made cuticle preparations and obtained soluble fractions. By Western blot analysis, we detected mNG-tagged proteins for both EPIC-1::mNG and EPIC-2::mNG in preparations of soluble cuticle proteins from mixed stages (Fig. S2A). These observations validate the expression of mNG-tagged EPIC proteins as components of cuticle.

### Loss of function in EPIC genes has mild effects on cuticle morphology and function

**Fig. 4.**
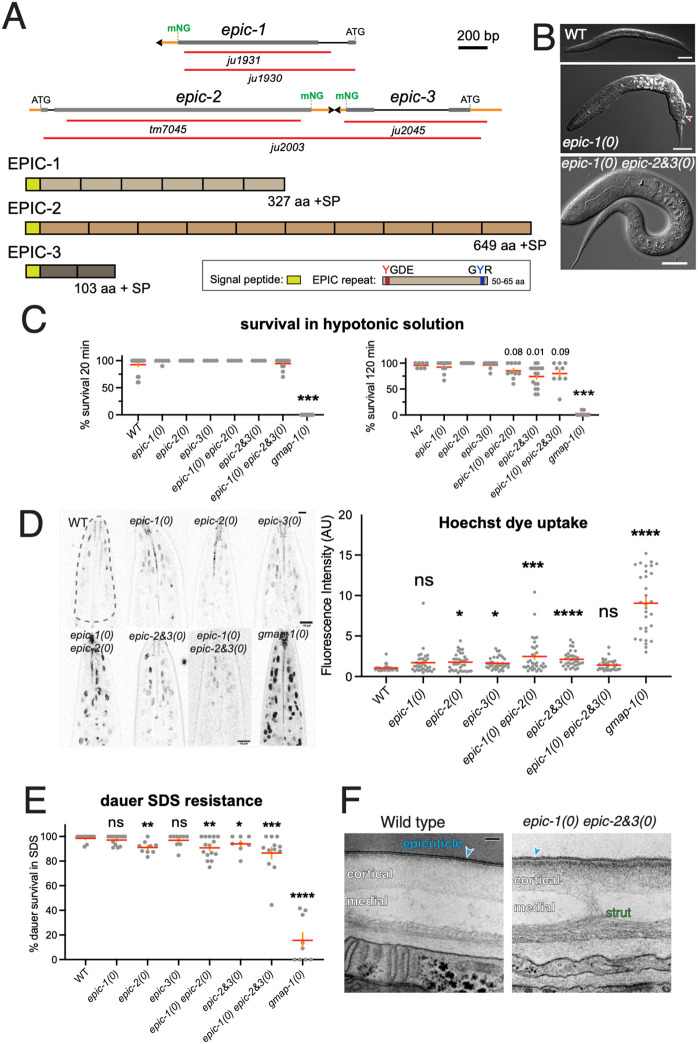
**EPIC mutants and phenotypes.** (A) EPIC gene structures, and schematics of EPIC proteins and the epicuticlin repeats (brown boxes). Signal peptides (SP) are indicated by yellow boxes; the number of residues indicated is after SP cleavage. (B) L1 larval morphology in wild type and examples of low penetrance phenotypes observed in *epic-1(ju1930)* [*epic-1(0)*] and in *epic-1(ju1930) epic-2&3(ju2003)* [*epic-1(0) epic-2&3(0)*] strains. An example (arrowhead) of tail morphology defects and blocked rectum from *ju1930*. The bottom image provides an example of a L1 arrested *epic-1(ju1930) epic-2&3(ju2003)* animal. Scale bars: 20 µm. (C) Hypotonicity survival assays in wild type and EPIC mutants, showing the fraction alive at 20 min and 120 min. Permeability barrier defective mutants, such as *gmap-1(0)*, were 0% viable (ruptured) after 20 min (*P*<0.001 compared with N2, Kruskal–Wallis test). EPIC mutant survival was reduced at 120 min, although this was only significantly different from N2 for *epic-2&3(ju2003)* (*P*=0.01); other groups are not significant unless indicated. *n*=8-10 sets of 10 animals per time point. (D) Hoechst uptake assay: confocal images of head regions. Scale bars: 10 µm. Region of interest for quantitation is outlined for wild type. A Kruskal–Wallis test and Dunn's multiple comparison test were used to test for significance. ns, not significant; *P<0.05; ***P<0.001; ****P<0.0001. (E) Dauer survival after SDS treatment. Each data point represents a trial of 10-20 dauer larvae. The fraction surviving was pooled across assays for comparisons to N2 using Fisher Exact test; *P<0.05; **P<0.01; ***P<0.001; ****P<0.0001. (F) TEM images of epicuticle in wild-type and *epic-1(0) epic-2&3(0)* adults. Images are representative of two wild type and four EPIC triple mutants (day 1 adults). The epicuticle (blue arrowheads) in *epic-1(0) epic-2&3(0)* animals was 6-14 nm thick, compared with 8.5-15 nm in wild type; the cortical layer beneath the epicuticle was consistently more darkly stained in EPIC mutants versus wild type.

We generated EPIC gene deletion mutations by CRISPR/Cas9 mediated gene editing and examined available deletion alleles generated by knockout projects (Fig. 4A; Table S1). We generated two deletions of *epic-1* using CRISPR/Cas9 (see Materials and Methods). *epic-1(ju1930)* mutants were viable and fertile, as were *epic-1(ju1931)* mutants; below, we focused on *epic-1(ju1930)* as a likely molecular null mutant. *epic-2(tm7045)* deletes repeats 2-10 and causes a frameshift in repeat 1. To assess the effects of *epic* partial deletion mutations, we assayed transcripts by RT-PCR (Fig. S2B); owing to the repetitive sequence nature of *epic-1* and *epic-2*, RT-PCR of *epic-1* or *epic-2* generated multiple bands in wild-type animals. *epic-2(tm7045)* mutants expressed truncated transcripts (Fig. S2B). *epic-2* and *epic-3* are immediately adjacent in tail-to-tail orientation; we generated a deletion eliminating most of both genes, *epic-2&3(ju2003)*, as well as a deletion eliminating *epic-3* alone, *ju2045*. All the above mutants were viable and fertile, with low penetrance lethality (Table S1). Most animals displayed a wild-type body shape and cuticle morphology by DIC microscopy; rare morphological defects were observed, such as tail morphology defects and blocked rectal regions. To investigate potential functional redundancy between *epic-1* and *epic-2* or *epic-3*, we generated double and triple mutants by recombination. *epic-1(ju1930) epic-2(tm7045)* and *epic-1(ju1930) epic-2&3(ju2003)* compound mutants were viable and fertile with normal cuticle morphology (e.g. alae, annuli and furrows, as assessed by DIC) and rare embryonic or L1 arrest (Fig. 4B).

**Fig. 5.**
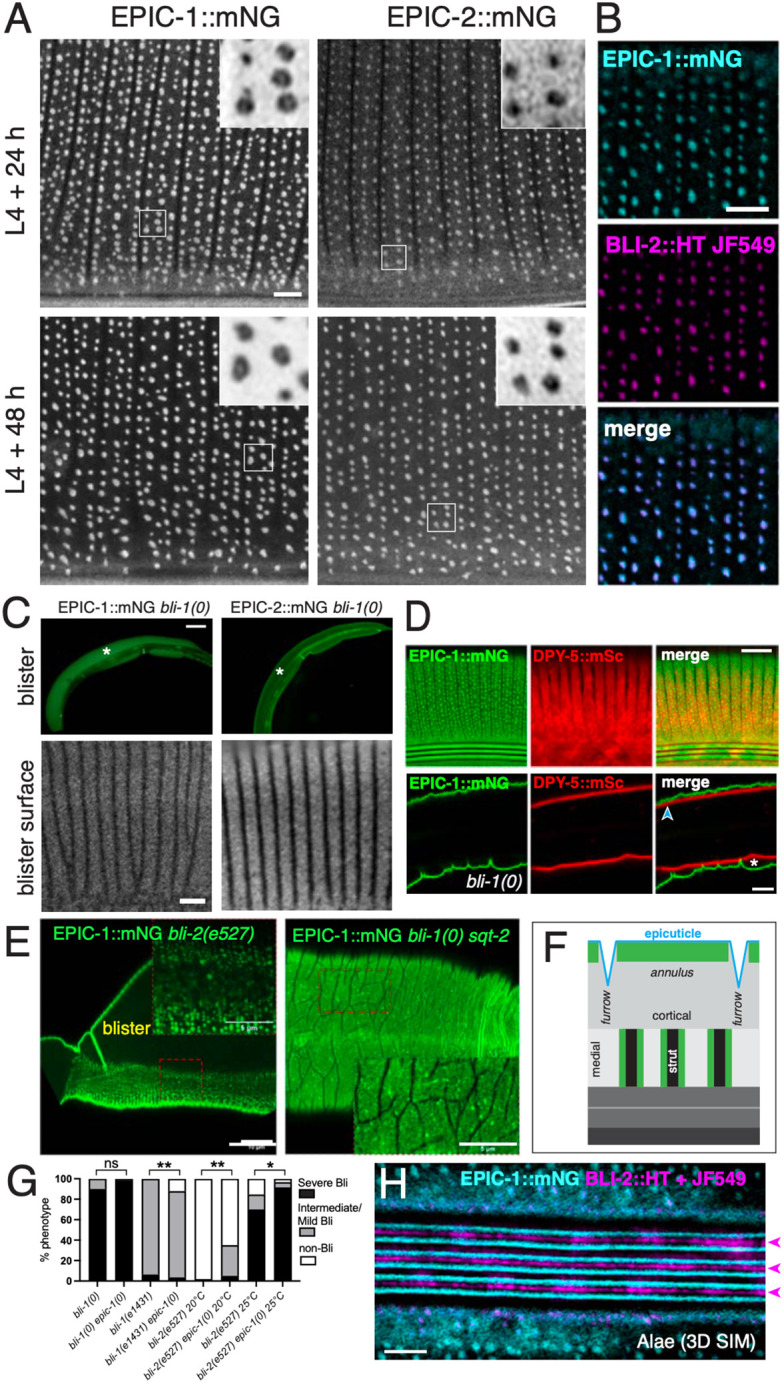
**Localization of EPIC proteins to adult struts and alae, and interactions with BLI collagens.** (A) EPIC-1::mNG and EPIC-2::mNG in adults 24 h and 48 h post-L4 showing anterior body lateral epidermis using Airyscan. Scale bar: 2 µm. Insets showing donut morphology (more often seen for EPIC-1::mNG) are 2×2 µm. (B) Colocalization of EPIC-1::mNG (cyan) and BLI-2::HaloTag (HT) stained with JF549 dye (magenta) in 3D SIM using 3D SIM, single focal plane. Scale bar: 2 µm. (C) EPIC-1::mNG and EPIC-2::mNG in *bli-1(ju1395)* null mutant adults: low magnification wide-field views showing accumulation in blisters; high magnification view showing localization to cortical annuli on surface of blister. EPIC-1::mNG localization to vulva or rectum was not affected in *bli-1(0)*. Scale bar: 50 µm (top panels); 2 µm (bottom panels). (D) Double labeling of EPIC-1::mNG with the fibrous layer annular marker DPY-5::mSc in wild-type and *bli-1(0)* adult. The latter is single deep focal plane showing separation of EPIC-1::mNG and DPY-5::mSc at blisters (asterisk) but not at unblistered regions (blue arrowhead). (E) EPIC-1::mNG localization in *bli-2(e527)* partial loss-of-function mutants and in *bli-1(0) sqt-2(sc3)* [*bli-1(0) sqt-2*] suppressed animals. In blistered regions of *bli-2(e527)* at 25°C, EPIC-1::mNG diffusely localized to the cortex; in non-blistered regions of *bli-2(e527)*, EPIC-1::mNG formed puncta, many of which had ‘donut’ morphology. In *bli-1(0) sqt-2(sc3)* animals where blistering is suppressed in the absence of struts, EPIC-1::mNG diffusely localized in the cortex with small scattered puncta that lacked donut morphology. (F) Model of adult EPIC-1::mNG localization at the cortical surface and at struts. (G) Genetic interactions of EPIC mutants with Bli mutants. Blistering (Bli) was scored 24 h post mid-L4 stage at 20°C or 25°C (see Materials and Methods). Fisher's exact test was used to test for statistical significance. ns, not significant; *P<0.05; **P<0.01. (H) BLI-2::HT stained with JF549 (magenta) localized to adult alae ridges (arrowheads) and EPIC-1::mNG (cyan) localized to six lines along the sides of ridges. Image is a maximum intensity projection 3D SIM. Scale bar: 2 µm.

We used RT-PCR to examine whether EPIC transcript levels displayed compensation (Fig. S2B). *epic-1* transcripts were expressed at normal levels in *epic-2(tm7045)* and in *epic-2&3(ju2003)*. Conversely, *epic-2* transcripts appeared normal in *epic-1(ju1930)*, although it was not possible to establish a quantitative difference due to variable priming from internal repeats. Based on these data, EPIC transcripts do not display significant compensation. EPIC-1::mNG localization in *epic-2(tm7045)* appeared indistinguishable from normal, as did EPIC-2::mNG localization in *epic-1(ju1930)* (Fig. S2C), suggesting EPIC-1 and EPIC-2 do not regulate the protein levels or localization of one another.

We further assessed cuticle permeability barrier function in EPIC mutants and observed mild cuticle permeability defects compared with barrier function mutants such as *gmap-1* (Njume et al., 2022). For example, in assays of survival in hypotonic solution, 70-80% of *epic-1(0) epic-2&3(0)* or *epic-2&3(0)* mutants were viable after 120 min compared with 95% of wild type or 0% of *gmap-1(0)* mutants (*n*>100 per genotype, Fig. 4C). We further assayed permeability barrier function using Hoechst 33342 dye uptake (Fig. 4D) and found EPIC mutant adults generally displayed mildly elevated Hoechst dye uptake compared with wild type, although significantly less than *gmap-1(0)* animals (Fig. 4D). Hoechst uptake was most increased in *epic-1(0) epic-2(0)* and *epic-2&3(0)* double mutants; *epic* triple mutants displayed a slight increase in Hoechst uptake that was not statistically significant. EPIC single mutant dauer larvae appeared morphologically normal and displayed normal or slightly reduced levels of SDS resistance; EPIC double mutants displayed significantly reduced resistance, and EPIC triple mutants displayed the strongest defects (Fig. 4E). Although these phenotypes were milder than those of barrier mutants such as *gmap-1(0)*, they suggest EPIC proteins have partly redundant roles in dauer cuticle function. At the ultrastructural level, the epicuticle of *epic-1(0) epic-2&3(0)* animals was slightly but significantly thinner than in the wild type (9.6±1.6 nm versus 11.9±1.9 nm in wild type, mean±s.e.m.; *P*=0.0017, *t*-test, *n*=15; Fig. 4F); otherwise, cuticle morphology appeared normal. Our results suggest that loss of individual or multiple EPIC proteins results in partial but significant reduction in permeability barrier function without major changes in aECM structure.

### EPIC-1 and EPIC-2 localize to adult struts, dependent on BLI collagens

Struts are adult-specific columnar structures that connect cortical and basal cuticle layers, spanning the fluid-filled medial layer (Adams et al., 2023). Loss of function in strut collagens leads to separation of cuticle layers (‘blistering’, the Bli phenotype). Although EPIC mutants did not display overt blistering, EPIC-1::mNG and EPIC-2::mNG both displayed adult-specific puncta resembling strut puncta, as defined by the BLI collagens (Fig. 5A). The punctate localization of EPIC-1::mNG and EPIC-2::mNG was in addition to their diffuse localization in cortical cuticle; in confocal *z* series, EPIC-1::mNG or EPIC-2::mNG puncta were much brighter than the diffuse cortical fluorescence seen in younger adults, suggesting they may reflect new EPIC protein synthesis in the adult.

EPIC-1::mNG and EPIC-2::mNG proteins were expressed through L4.5-L4.9 but remained diffuse in the cortical cuticle and were not localized to struts (Fig. S3A). By examining staged adults, we found that EPIC-1::mNG and EPIC-2::mNG became recruited to struts beginning ∼12 h after the L4/adult molt. These observations indicate the EPIC proteins are recruited to struts in early adult life. EPIC-1::mNG and EPIC-2::mNG could also be visualized in the epidermal secretory pathway (Fig. S3B) until at least 48 h in adulthood, suggesting EPIC proteins are secreted by the epidermis in adults.

We focused on EPIC-1::mNG for quantitative analysis because EPIC-1::mNG displayed a clearer transition from diffuse to punctate compared with EPIC-2::mNG. Patterning of EPIC-1::mNG puncta resembled those of BLI-1 or BLI-2 puncta, in that they typically formed three circumferential rows per annulus (two furrow-flanking rows and a more variable central row) (Fig. 5A), with spacing of 0.81±0.07 µm (mean±s.d., *n*=10 rows), compared with 0.77 µm spacing for BLI-1::mNG. The density of EPIC-1::mNG puncta in midbody lateral cuticle was 153 puncta per 100 µm^2^ (*n*=6 ROIs) compared with 145-183 puncta per 100 µm^2^ for BLI-1::mNG (Adams et al., 2023).

We have previously shown that struts contain the three BLI collagens BLI-1, BLI-2 and BLI-6. We found that EPIC-1::mNG and BLI-2::HaloTag (HT) colocalized in adult struts (Fig. 5B). EPIC-1::mNG and BLI-1::mSc also displayed significant colocalization in adults but not in L4 (Fig. S3C). EPIC-1::mNG and BLI-1::mSc displayed a mean Pearson colocalization coefficient of +0.03 at L4+12 h before EPIC-1 puncta formation, increasing to +0.52 at L4+24 h after EPIC puncta formation (*n*=6 ROIs per time point, Fig. S3C,D), similar to the degree of colocalization of BLI-1::mSc and BLI-2::mNG (Adams et al., 2023).

Our 3D structured illumination microscopy (SIM) analysis of BLI-1 and BLI-2 knock-ins revealed that both collagens show cylindrical localization in struts, appearing donut-shaped in single *z* cross-sections (Adams et al., 2023). We performed 3D SIM on EPIC-1::mNG in adult stages and observed donut-shaped structures at struts; however, the SIM reconstruction quality was low due to the diffuse localization of EPIC-1::mNG signal in the cortical layer. We therefore estimated EPIC donut size from Airyscan images (Fig. 5A). In line scans of EPIC-1::mNG puncta displaying donut morphology, meaning a central minimum surrounded by peaks, EPIC-1::mNG peak to peak diameter was 239±47 nm in adults 24 h post-L4 (mean±s.e.m., *n*=11) and 280±63 nm in adults 48 h post-L4 (*n*=11) compared with our SIM measurements of BLI-1::mNG peak to peak diameter of 160 nm (Adams et al., 2023). These observations suggest the EPIC donuts may be slightly larger in diameter than the BLI donuts, consistent with EPIC proteins being recruited to the outer layer of struts.

We next addressed whether EPIC::mNG localization in struts depended on BLI collagens. In *bli-1(ju1395)* null mutants that lack struts and have an expanded medial layer (‘blister’) (Adams et al., 2023), EPIC-1::mNG formed a diffuse granular pattern in annular bands at the outer surface of the blister and did not form strut-like puncta (Fig. 5C); EPIC-1::mNG localization to alae or interfacial cuticle was not affected. EPIC-2::mNG was also localized to the blister surface in *bli-1(0)* (Fig. 5C). Both EPIC-1::mNG and EPIC-2::mNG variably accumulated within the fluid blister. Double labeled DPY-5::mSc EPIC-1::mNG adults showed separation of the cortical and basal layers within blisters (Fig. 5D, asterisk). Together, these observations suggest that EPIC proteins require struts for their punctate localization but not for their cortical localization. We further examined EPIC-1::mNG localization in the partial loss-of-function allele *bli-2(e527*ts*)*. In this background, EPIC-1::mNG formed fewer puncta that ranged from normal donut-like morphology to smaller puncta (Fig. 5E). In *bli-1(0) sqt-2(sc3)* double mutants, blistering is suppressed but struts are largely absent; in such animals, EPIC-1::mNG formed occasional small puncta (Fig. 5E), reminiscent of BLI-2::mNG puncta in this background (Adams et al., 2023). Taken together, these observations indicate that reduced BLI-1 or BLI-2 results in reduced EPIC-1 recruitment to struts. In other cuticle collagen mutants such as *dpy-3*, *dpy-5* or *sqt-2*, EPIC-1::mNG puncta were aberrantly patterned (Fig. S3E), correlating with strut pattern disruption in these mutants (Adams et al., 2023). Conversely, BLI-1 in *epic-1(0)* or *epic-2(0)* single mutants, and in *epic-1(0) epic-2(0)* double mutants was normal (Fig. S3F). Together, these data suggest that in adults EPIC-1::mNG displays dual localization to struts, as well as to the cortical compartment (see schematic, Fig. 5F).

*epic-1(0)* did not detectably enhance or suppress the Bli phenotypes of *bli-1(0)* null mutants (Fig. 5G). Moreover, *bli-1(0) epic-1(0)* mutants displayed similar phenotypic progression as *bli-1(0)* mutants, with 19/41 double mutants becoming severely blistered within 7 h of mid L4 stage compared with 24/40 *bli-1(0)* mutants. *epic-1(0) bli-1(e1431)* double mutants displayed partial suppression of the *e1431* intermediate Bli phenotype from 100% to 88% penetrance. In contrast, *epic-1(0)* significantly enhanced *bli-2(e527)* partial loss of function: at 20°C, *bli-2(e527)* mutants were 2% blistered, whereas *bli-1(e527) epic-1(0)* animals were 35% blistered. Overall, these results suggest loss of EPIC-1 function can modify Bli mutant phenotypes in different ways; below, we discuss possible reasons for these complex genetic interactions.

Although diffuse localization of EPIC-1::mNG precluded 3D SIM reconstructions in struts, we were able to use 3D SIM to visualize EPIC-1::mNG and BLI-2::HT at adult alae. BLI-2::HT formed three longitudinal stripes corresponding to alae ridges, whereas EPIC-1::mNG could be resolved into six longitudinal stripes flanking the ridges (Fig. 5H). EPIC-1::mNG also localized to diffuse longitudinal bands flanking the alae. These observations indicate that BLI-2 and EPIC-1 localize to distinct parts of the adult alae.

### Cortical EPIC-1::mNG localizes close to the epicuticle lipid layer

**Fig. 6.**
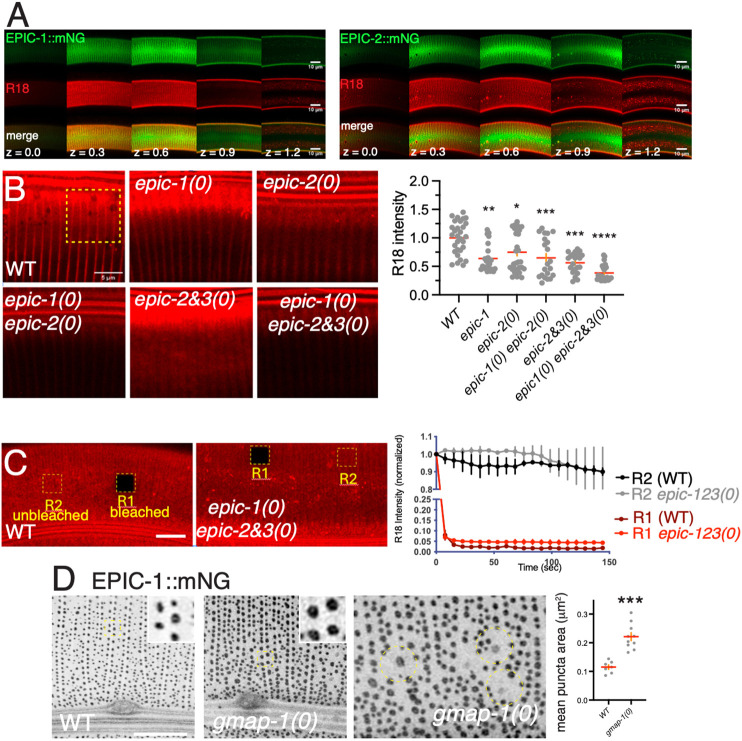
**Localization of EPIC proteins with lipophilic dyes, and interactions with the lipid transport protein gene *gmap-1*.** (A) Montages of R18 staining (red) in EPIC-1::mNG and EPIC-2::mNG. Images are 5×0.3 µm thick Airyscan planes from cortical to basal in an L4 stage larva (*z* dimensions in μm). EPIC-1::mNG and R18 colocalize at *z*=0.3 and 0.6; EPIC-1::mNG and EPIC-2::mNG also localize at *z*=0.9, distinct from R18. *xy* scale: 10 µm. (B) R18 staining in wild type (N2) and EPIC mutant adults (L4+24 h). Images are maximum intensity projections of 5×0.3 µm focal planes of R18 cuticle staining over lateral epidermis, with the central *z* plane in the middle of the alae (top of images). Scale bar: 5 µm. Quantitation of mean R18 intensity in 10 µm^2^ regions of interest (yellow dashed box) of MIPs (three per animal, 7-10 animals per genotype). A Kruskal–Wallis test was used to test for statistical significance; *P<0.05; **P<0.01; ***P<0.001; ****P<0.0001. Data are mean±s.e.m. (C) Representative FRAP of R18 in N2 and EPIC triple mutant adults. ROI1 (R1) is bleached; R2 is control unbleached. Scale bar: 10 µm. Graph shows R18 fluorescence normalized to pre-bleach intensity (arbitrarily 1). Data are mean±s.e.m. *n*=3 experiments per genotype. No recovery was observed in N2 or in EPIC triple mutants. (D) EPIC-1::mNG puncta morphology in wild-type adult and *gmap-1(ulb13)* null mutants. Anterior epidermis showing anterior deirid sensilla and alae. Right panel shows an example of EPIC-1::mNG ‘crop circles’ in *gmap-1(0)* mutants (circular dashed outlines). Scale bar: 10 µm, insets are 2×2 µm. EPIC-1::mNG puncta size was significantly increased in *gmap-1(0)*. A two-tailed unpaired Student's *t* test was used to test for statistical significance, ***P<0.001.

Our confocal and TIRF imaging indicated that EPIC-1::mNG localized to an outer layer of cuticle. To assess its localization relative to the epicuticle, we stained EPIC-1::mNG animals with the lipophilic dye R18 (see Materials and Methods) (Proudfoot et al., 1993). R18 normally stains annular ridges and furrows; in *z* stacks, the topmost R18 signal could be detected in two or three cortical sections of which one or two overlapped with diffuse EPIC-1::mNG and EPIC-2::mNG in L4 stage (Fig. 6A, orthogonal sections); in adults, BLI-1::mNG did not overlap with R18 staining (Fig. S4A). We further examined R18 and EPIC-1::mNG colocalization in TIRF. The outermost R18 and EPIC-1::mNG signals were observed under stringent TIRF conditions (Fig. S4B), suggesting they colocalize within the level of *z*-resolution of TIRF. R18 epicuticle staining was significantly reduced in EPIC mutants but was otherwise similar in distribution (Fig. 6B).

Epicuticle lipids show low lateral mobility in other nematodes, based on FRAP experiments (Kennedy et al., 1987). We assessed this in *C. elegans* using R18 staining and found no recovery over 2 min after photobleaching in the wild type, or in EPIC single, double or triple mutants (Fig. 6C; Fig. S4C), suggesting EPIC proteins do not strongly affect lipid lateral mobility. Conversely, EPIC-1::mNG fluorescence did not recover after photobleaching (Fig. S4D), suggesting EPIC-1 is stably localized within the aECM.

The GM2AP-like lipid transporter GMAP-1 is implicated in epicuticle lipid biogenesis and barrier function (Njume et al., 2022). In *gmap-1(ulb13)* null mutants, EPIC-1::mNG puncta were larger than in wild type (Fig. 6D). The fraction of a region of interest (ROI) occupied by puncta was significantly increased (24% versus 16.9% in wild type, *P*=0.0003 by Student's *t*-test, *n*=9 ROIs) as was punctum area (Fig. 6D). *gmap-1(0)* mutants also displayed scattered ‘crop circles’ containing a larger less intense EPIC-1::mNG punctum surrounded by a region ∼2 µm diameter lacking EPIC-1::mNG puncta (Fig. 6D, circled). These observations suggest the diffuse localization of EPIC-1::mNG is not significantly affected by loss of *gmap-1*; cuticle lipids may affect strut morphology or EPIC distribution at struts.

### EPIC proteins localize to wound scars and contribute to post-wounding survival

**Fig. 7.**
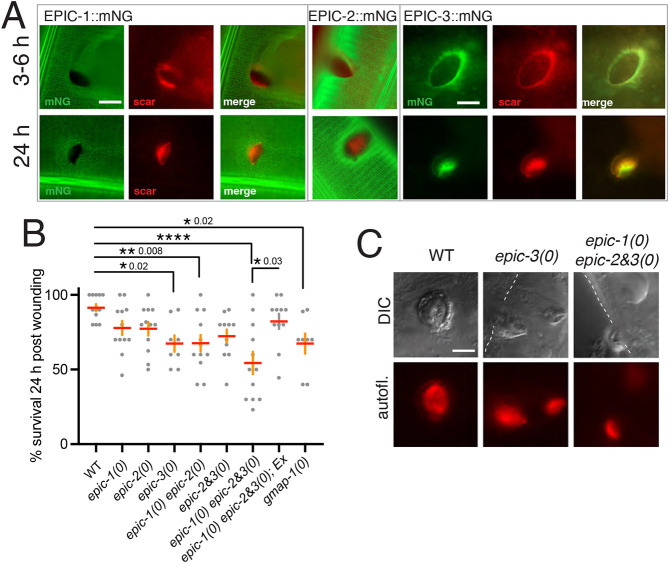
**EPIC proteins and epidermal wound repair.** (A) EPIC-1::mNG, EPIC-2::mNG and EPIC-3::mNG localization at wound sites 3-6 h and 24 h post-needle wounding. Needle wounding results in a 5-10 µm diameter hole in the adult EPIC-1::mNG pattern with an autofluorescent scar (red channel) by 3-6 h post-injury. EPIC-1::mNG occasionally showed diffuse localization around the wound site but did not colocalize with scar autofluorescence (red channel). EPIC-2::mNG after wounding overall resembled EPIC-1::mNG (merged images shown for 3 h and 24 h). EPIC-3::mNG localized to wounds at 6 h and closed puncta at 24 h, often colocalizing with scar autofluorescence. Images are widefield. Scale bars: 10 µm. (B) Survival of EPIC mutants post-wounding. Each point represents 10 animals. One-way ANOVA and Dunnett's multiple comparisons test were used to test for significance. Comparisons with N2 wild type are not significant unless indicated; *P<0.05; **P<0.01; ****P<0.0001. *epic-1(0) epic-2&3(0); Ex* denotes *epic-1(+)* transgenics. (C) Wound scars in wild type, *epic-3(0)* and *epic-1(0) epic-2&3(0)* triple mutants at 24 h post-wounding. Images are DIC and widefield. Scale bar: 5 µm. In EPIC mutants, scars were fragile and fragmented or partially detached from the cuticle surface (dashed lines) after slide mount.

*epic-3* expression is upregulated after needle wounding (Fu et al., 2020; Yu et al., 2024). We therefore examined whether EPIC proteins may be involved in cuticle repair after wounding. EPIC-1::mNG and EPIC-2::mNG showed occasional localization to rings around the wound site (Fig. 7A), whereas EPIC-3::mNG localized to rings by 6 h post-wounding; by 24 h, EPIC-3::mNG rings typically had contracted to puncta (Fig. 7A). As EPIC-3::mNG fluorescence was not detectable in unwounded adults, these results are consistent with *epic-3* transcriptional upregulation after wounding. We next examined survival of EPIC mutants 24 h after wounding. Survival of *epic-3(0)* single or double mutants was reduced, with *epic-1(0) epic-2&3(0)* triple mutants displaying the lowest survival; reduced survival of the triple EPIC mutant was partially rescued by expression of *epic-1(+)* (Fig. 7B). *gmap-1(0)* mutants also displayed reduced survival post-wounding, suggesting barrier function is crucial in wound repair (Fig. 7B). Needle wounding of wild-type animals creates disk-shaped refractile autofluorescent scars (Pujol et al., 2008); *epic-3(0)* and *epic-1(0) epic-2&3(0)* triple mutant scars resembled those in the wild type; however, these scars often became detached from the cuticle or fragmented (Fig. 7C), suggesting EPIC proteins are not essential for scar formation but may securely attach scars to surrounding cuticle.

## DISCUSSION

Lipid-rich extracellular layers are widely found in the aECM of barrier epithelia from invertebrates to humans, yet their organization and interaction with other aECM constituents remains little understood. Here, we focused on the *C. elegans* epicuticlins, and found they localize to specific aECM substructures close to the epicuticle. EPIC proteins also localize to other aECM substructures that may have specialized epicuticles. We find that loss of epicuticlin function has minimal effect on cuticle morphology but mildly impairs barrier function and wound repair, suggesting epicuticlins play accessory roles in the aECM.

### The *C. elegans* epicuticle and epicuticlins

Our analysis of available HPF fixed EM sections is consistent with classical TEM studies that have suggested the epicuticle resembles a thickened lipid bilayer (Cox et al., 1981a). Our measurements of epicuticle thickness are also within the range of thicknesses reported for other nematode epicuticles, which range from 6 to 40 nm (Bird, 1980). Our FRAP imaging of lipophilic dyes supports the model that *C. elegans* epicuticular lipids do not freely diffuse.

Our analysis of *C. elegans* EPIC expression supports their localization to the epicuticle and/or the underlying external cortical layer, corresponding to the insoluble fraction of the cuticle, and broadly consistent with immunoelectron microscopy studies in parasitic nematodes (Bisoffi et al., 1996). EPIC proteins are made up of low-complexity repeats with a biased amino acid composition. EPIC-1 and EPIC-2 together contain ∼34.5% alanine, excluding their signal peptides. In contrast, other proteins in the insoluble fraction, such as CUT proteins, are <10% Ala, with the exception of the tandem repeat protein CUT-2 (27% Ala) (Lassandro et al., 1994). EPIC proteins might contribute to the biased amino acid composition of the insoluble cuticle fraction (19.5% Ala versus 11% in the soluble fraction).

EPIC::mNG knock-in proteins localize to an external cortical layer of the cuticle, close enough to the surface that they can be visualized using true TIRF microscopy. Owing to the thickness of the cuticle or eggshell, most *C. elegans* studies have used near-TIRF/semi-TIRF with penetration depths of ∼500 nm (Robin et al., 2014). Our ability to detect cortical EPIC-1::mNG signal under true TIRF conditions indicates the outermost EPIC-1 signal is within 100 nm of the surface of the animal, consistent with localization to the epicuticle or external cortical layer. The labyrinthine patterns of EPIC-1::mNG in furrow collagen mutant backgrounds are reminiscent of cuticle surface topography of such mutants under atomic force microscopy (Aggad et al., 2023), further supporting the localization of EPIC-1 near the cuticle surface. In larvae, the EPIC-1::mNG and EPIC-2::mNG proteins are localized in annular ridges, whereas lipophilic dyes stain annuli and furrows, suggesting EPIC proteins do not uniformly underpin the lipid layer. Loss of function in one or more EPIC genes resulted in partial but consistent defects permeability barrier function, in contrast to the severe defects of mutants such as *gmap-1(o)*. These observations suggest EPIC proteins may play an accessory role in permeability barrier function.

### EPIC proteins localize to multiple aECM compartments

EPIC proteins are also highly expressed in interfacial cuticle areas. Cuticle overlying interfacial epithelial cells or glial cells has a distinct composition (Fung et al., 2023; Fung et al., 2024). The ultrastructure of the epicuticle or its boundaries have not been extensively characterized in *C. elegans*. In other nematodes, the epicuticle terminates within interfacial regions (Dick and Wright, 1974; Bird, 1980). Based on public EM datasets, *C. elegans* does not appear to generate occluding cuticle plugs, as seen in some other nematodes (Vincent et al., 1979); however, EPIC proteins may play roles in reinforcing the epicuticle at boundaries. Interestingly, EPIC proteins localize to the dauer buccal plug, which is the thickened cuticle that seals the mouth. Little is known about the biogenesis or composition of the buccal plug; however, it has a highly osmiophilic luminal surface in TEM (Albert and Riddle, 1983) that may be related to the body epicuticle.

Unexpectedly, EPIC-1 and EPIC-2 both localized to struts beginning in adulthood. These observations suggest that struts undergo adult maturation, being initially composed of the collagens BLI-1, BLI-2 and BLI-6, and later recruiting EPIC-1 and EPIC-2. Molecular epistasis indicated that BLI proteins are required for EPIC proteins to localize to struts and not the reverse. We find that EPIC proteins also display nanoscale organization: under similar Airyscan parameters, BLI-1::mNG donuts were not resolvable, whereas EPIC-1 donuts were consistently resolvable, suggesting EPIC-1 may be recruited to the outside of BLI-1-containing struts.

EPIC single, double and triple mutants did not display adult blistering (Bli) phenotypes. *epic-1(0)* did not enhance the phenotypes of *bli-1* null mutants, yet it enhanced the Bli phenotypes of *bli-2(e527)* partial loss of function at the permissive temperature, suggesting that EPIC-1 has a cryptic role in strut function. Conversely, *epic-1(0)* partially suppressed the Bli phenotypes of *bli-1(e1431)*, which has intermediate BLI-1 function. This may parallel the suppression of *bli-1(e769)* Bli phenotypes by *gmap-1(0)*, as a permeability barrier is required to maintain the fluid-filled medial layer in blisters (Njume et al., 2022). The ability of *epic-1(0)* to enhance or suppress hypomorphic Bli phenotypes may reflect its dual roles in barrier and strut function. Reduced barrier function may be insufficient to suppress the effects of complete absence of struts in *bli-1(0)* null mutants.

### Epicuticlins and tandem repeat proteins in the aECM

Many aECM proteins are composed of imperfect tandem repeats, such as elastins (He et al., 2007), gel-forming mucins (Perez-Vilar and Hill, 1999) or silk spidroins (Baker et al., 2022). Epicuticlins are distinctive in containing perfect repeats. Other repetitive intrinsically disordered proteins have been identified in *C. elegans* aECMs, such as the chitinous pharynx cuticle (Kamal et al., 2022), suggesting aECMs may involve networks of intrinsically disordered proteins. Relative to imperfect repeats, perfect repeats tend to be unstructured (Jorda et al., 2010) and are under-represented in protein structure databases. The structures of epicuticlins are a challenge for future investigation.

Several vertebrate epidermal proteins, such as involucrin, filaggrin or trichohyalin, are composed of low-complexity tandem repeats and together form the cornified envelope (CE). Defects in the CE cause skin pathologies; for example, mutations in filaggrin cause ichthyosis vulgaris and predispose to atopic dermatitis (Sandilands et al., 2009). Genetic deletion of CE components such as involucrin has subtle phenotypic consequences in mice (Djian et al., 2000), although compound mutants display barrier defects (Sevilla et al., 2007), suggesting that mammalian epidermal CE components form a redundant network. Analogously, *C. elegans* EPIC proteins appear to act collectively in barrier function and in wound repair. In conclusion, the EPIC proteins define specific cortical compartments in the aECM with roles in barrier function. Our work also underscores the insights that can be gained from analysis of less-studied proteins (Perdigão et al., 2015; Rocha et al., 2023).

## MATERIALS AND METHODS

### General methods

*C. elegans* maintenance followed standard procedures; mutations were confirmed by PCR or sequencing. Strain genotypes, oligonucleotide sequences and details of new alleles used in this study are in Tables S2-S4.

### EPIC deletions and knock-in mutations

*epic-2(tm7045)* was generated by the Japanese National Bioresource Project and obtained in strain FX7045 from the laboratory of Shohei Mitani (Tokyo Women's Medical University School of Medicine, Japan). *epic-1(gk961616)* was generated by the *C. elegans* Million Mutation Project (Thompson et al., 2013) and obtained in strain VC40784 from the CGC. These deletions were outcrossed to N2 two or three times before analysis. The *gk961616* background displayed higher levels of lethality not observed in *epic-1(0)* mutants and was not pursued further. We used the melting method (Ghanta and Mello, 2020) to generate larger deletions in *epic-1* and isolated two deletion alleles, *ju1930* and *ju1931* (Table S1). To create *epic-2* and *epic-3* compound mutants we performed CRISPR in the *epic-2(tm7045)* background and isolated three deletions *ju2003*, *ju2004* and *ju2005*; *ju2004* and *ju2005* were 1 bp smaller than *ju2003* and were not analyzed in detail. *epic-3(ju2045)* deletes the entire *epic-3*-coding sequence; a second identical, but independent, deletion was recovered as *ju2053*.

*epic-1*, *epic-2* and *epic-3* knock-in strains were generated by SunyBiotech (Fuzhou, China). All knock-ins contain mNeonGreen inserted at the C-terminus with a 3xGAS linker. All three knock-in strains were viable and fertile, and displayed normal permeability barrier function. In strain constructions with other cuticle mutants, we did not detect enhancement or suppression of Dpy or Bli phenotypes by the EPIC knock-in alleles. The *dpy-5::mSc(syb3326)* knock-in is tagged with wrmScarlet at the C-terminus and was purchased from SunyBiotech.

### Transgenic rescue

We generated a 1.7 kb genomic fragment containing the *epic-1* gene by Phusion PCR from N2 DNA template using primers SD21418 and SD21419. Transgenes were generated by co-injection of PCR product (5 ng/µl) and the P*inx-6*-RFP marker pAB1 (100 ng/µl) using standard procedures. Three highly transmitting arrays were selected; the rescue data in Fig. 7B show *juEx8461*.

### RT-PCR

Mixed-stage animals were collected from large NGM plates, washed three times with M9 and incubated for half an hour with rotation at room temperature. After washing, worm pellets were collected by centrifugation at 2000* **g*** and frozen overnight at −80°C in 1 ml of TRIzol reagent (Invitrogen). RNA was isolated and solubilized in 25 µl DEPC-H_2_O and the concentration measured using a spectrophotometer. 10 µg RNA was treated with 1 µl DNAse (Invitrogen TURBO kit) at 37°C for 30 min, purified using the phenol chloroform method and precipitated using 100% RNA grade ethanol overnight at −20°C. Final pellets after purification were solubilized in 20 µl RNAse-free water, the concentration was measured and 1 µg RNA converted to cDNA using ThermoFisher Superscript III RT Kit. The resulting cDNA was used for PCR using cDNA specific primers (Table S3).

### Biochemistry

Biochemical analysis was performed as previously described (Adams et al., 2023).

### Imaging and fluorescence recovery after photobleaching

Widefield fluorescence and DIC imaging were performed on a Zeiss Axioplan M2 imager as described previously (Adams et al., 2023). Conventional confocal imaging and FRAP were performed on a Zeiss LSM800 confocal microscope. Airyscan super-resolution imaging was performed on a Zeiss LSM900 confocal. Images of EPIC ‘donut’ structures used super resolution and Auto deconvolution filter strengths (6.5-7.5). Donut morphology was similar at deconvolution filter strengths down to 6.0; higher filter strengths generated structured noise artifacts throughout the image.

3D SIM was performed on a Cytiva OMX microscope using levamisole immobilization, as described previously (Adams et al., 2023). TIRF imaging was performed on the OMX SR microscope platform (Cytiva) in TIRF mode using an Olympus ApoN 60×/1.49 objective (APON60XOTIRF) and circular TIRF illumination (RingTIRF) (Ellefsen et al., 2015). The penetration depth of the evanescent wave was controlled via OMX software (OMX Acquire) and for images in Fig. 2 was set to maximal stringency, i.e. the shallowest angle before loss of signal, yielding *z* resolutions <100 nm. For the *z* series in Fig. S1B, we used the variable-angle TIRF mode in the OMX (Dobbie et al., 2011).

For imaging of EPIC::mNG strains in dauer stage, dauer larvae were generated by starvation. For imaging of adults, animals were aged at least 24 h from mid L4 stage.

HaloTag JF549 ligand staining for visualization of BLI-2::HT in SIM was performed as described previously (Adams et al., 2023). EPIC puncta distribution was quantitated from single focal planes of Airyscan processed images of adults 24 h post-L4 stage. To measure puncta spacing, we drew 10-15 µm line scans along furrow-flanking rows of puncta and counted peaks. To measure puncta density and percentage area, we used a brightness threshold of 56-70 and a particle size range of 0.001-10 µm^2^, in two or three ROIs (100-400 µm^2^ each) per image.

### Lipophilic dye staining of epicuticle

Lipophilic dyes were purchased from ThermoFisher. Dye staining followed protocols based on other lipophilic dyes (Schultz and Gumienny, 2012). In brief, healthy unstarved mixed-stage animals were washed into microcentrifuge tubes with M9 and 0.5% Triton X-100, then washed twice with M9. The desired concentration of lipid dye was added to worms in M9 and the animals incubated for 3 h at room temperature on a rotator. Animals were washed two to four times with M9 then allowed to destain on an NGM agar plate for 10-30 min before imaging.

For lipid dye staining of mNG-expressing strains, we used octadecyl rhodamine chloride R18 (Catalog O246) at a concentration of 1 µM, which consistently stained the epicuticle and filled sensory neurons. Under our conditions, R18 stained annuli, furrows and two to four longitudinal valleys in the adult alae. R18 annular staining was occasionally non-uniform with smaller intense patches of staining or larger less intensely staining patches. At a concentration of 0.1 µM, R18 filled sensory neurons but did not stain the epicuticle; at a concentration of 10-100 µM, R18 stained internal membranes.

### Electron microscopy

Larval EM datasets have been described previously (Witvliet et al., 2021). New L4 and adult samples were prepared as described previously (Aggad et al., 2023). Epicuticle or plasma membrane thickness was measured using the Plot Profile function in Fiji in line scans perpendicular to the cuticle plane. ‘Peak to peak’ thickness (i.e. ‘trough to trough’ in the EM image) is the distance between the midlines of the two most osmiophilic regions.

### Permeability barrier, wound healing and skin blistering assays

For the hypotonicity survival assay, adults were picked into drops of double-distilled H_2_O and scored every 10 min for rupture at the vulva. For Hoechst uptake assays, animals were incubated in 10 µg/ml Hoechst 33342 (Thermo Fisher) dissolved in M9 buffer for 45 min. At least three trials were performed per genotype and at least 10 animals imaged per trial using a LSM800 confocal microscope. Background fluorescence was subtracted and fluorescence quantitated in ROIs containing the head region shown in Fig. 4E; values were normalized to the N2 control in each trial. Dauer larvae SDS resistance assays were performed following standard procedures (Nika et al., 2016); dauer larvae generated by starvation were transferred to 100 µl of 1% SDS in 96-well plates for 10 min then returned to NGM agar plates and tested for viability by response to mechanical stimuli. At least 100 larvae were tested per genotype in trials of 10-20 larvae; a 30 min incubation in SDS yielded identical results. Needle wounding was performed as described previously (Xu and Chisholm, 2014) on animals 24 h after the L4 stage, in the mid-anterior or posterior lateral epidermis. All wounded animals were viable and motile immediately after wounding.

For quantitation of skin blistering in L4 animals aged 24 h, ‘severe’ blistering was defined as the entire body being encased in a blister; such animals are usually small, paralyzed and lay few, if any, eggs. *bli-1(ju1395)* null mutants are 100% severe. Intermediate blistering is defined as a normal-sized animal with one or more blisters that did not impair egg laying; *bli-1(e1431)* animals are >95% intermediate. Mild blistering is defined as one or more localized blistered areas, most frequently in the tail. For statistical comparisons, all the above categories were pooled.

### Statistical analysis and reproducibility

All statistical analysis was carried out using GraphPad Prism 10. All datasets were tested for normality and parametric or non-parametric tests were used accordingly. At least three biological replicates per strain were analyzed independently (e.g. more than three animals per genotype imaged). Confocal fluorescence images are representative of 5-10 images per condition acquired over at least three sessions. In graphs, dot plots show the mean (red line) and s.e.m. (orange error bar).

## Supplementary Material

10.1242/develop.204330_sup1 Supplementary information
