# Supplementary material for: C. elegans epicuticlins define specific compartments in the apical extracellular matrix and function in wound repair
Source: Development. 2024 Oct 23;151(21):dev204330. doi: 10.1242/dev.204330 (PMC11529277; doi:10.1242/dev.204330)
Supplement: Supplementary information [file develop-151-204330-s1.pdf]

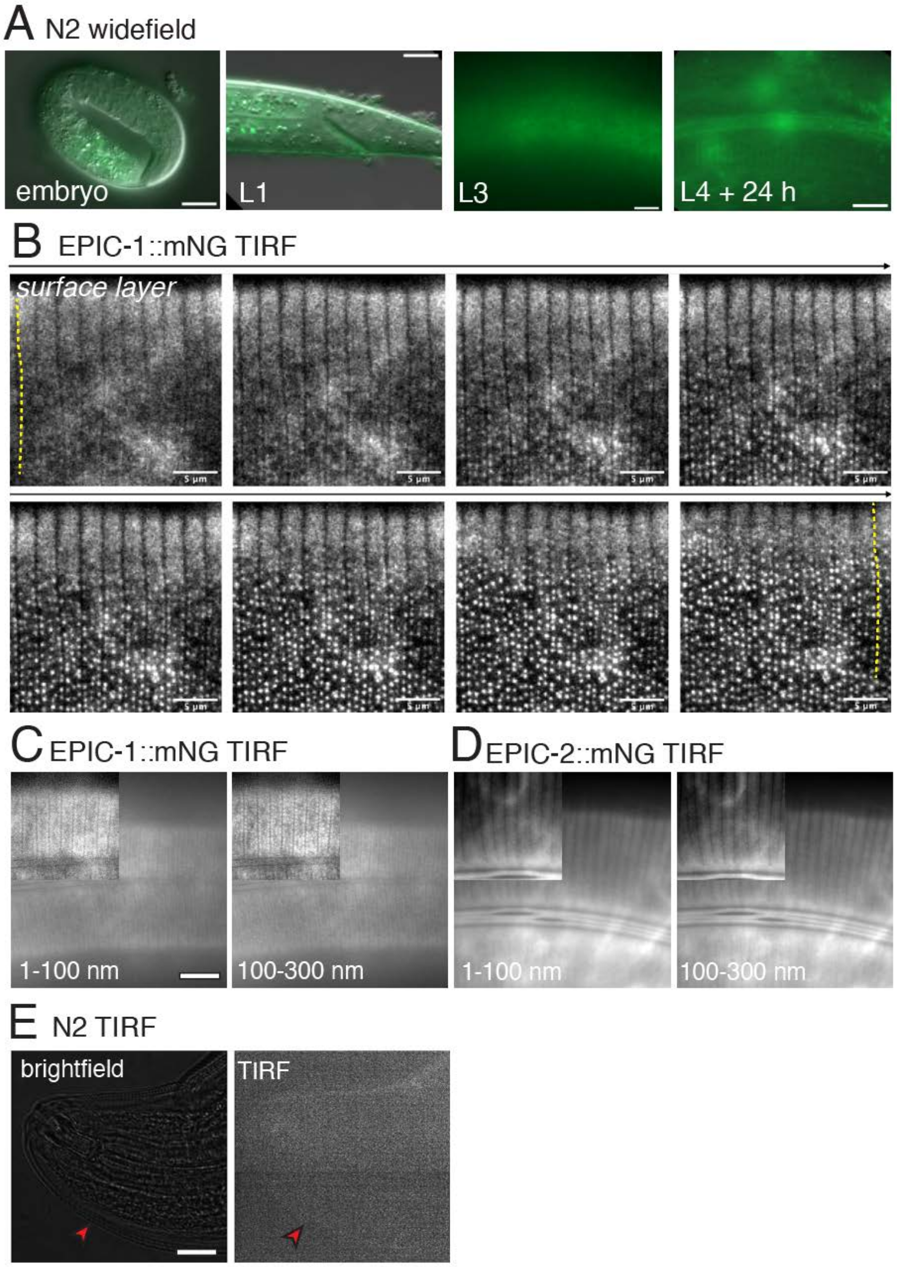

Fig. S1. Additional controls for EPIC imaging. A. N2 control fluorescence under DIC/widefield in embryos, larvae, and adults, showing minimal level cuticle autofluorescence. Scales, 10 µm. Setting were identical to those used in widefield images in Figure 2. B. EPIC-1::mNG in variable-angle TIRF microscopy of adult cuticle (L4+48 h), 8 successive z sections from external to internal in 125 nm steps. Cuticle furrow indicated by yellow dashed line. Scales, 5 µm. C,D. EPIC-1::mNG (C) and EPIC-2::mNG (D) in L4+24 h adult cuticle, raw TIRF images at maximum stringency showing background levels, insets show images after processing. Projections of outer 100 nm (2 x 50 nm slices) and next 200 nm (4 x 50 nm slices). Scales, 10 µm. E, N2 control imaged under brightfield and TIRF using same illumination conditions as Panels C,D. Scale, 10 µm.

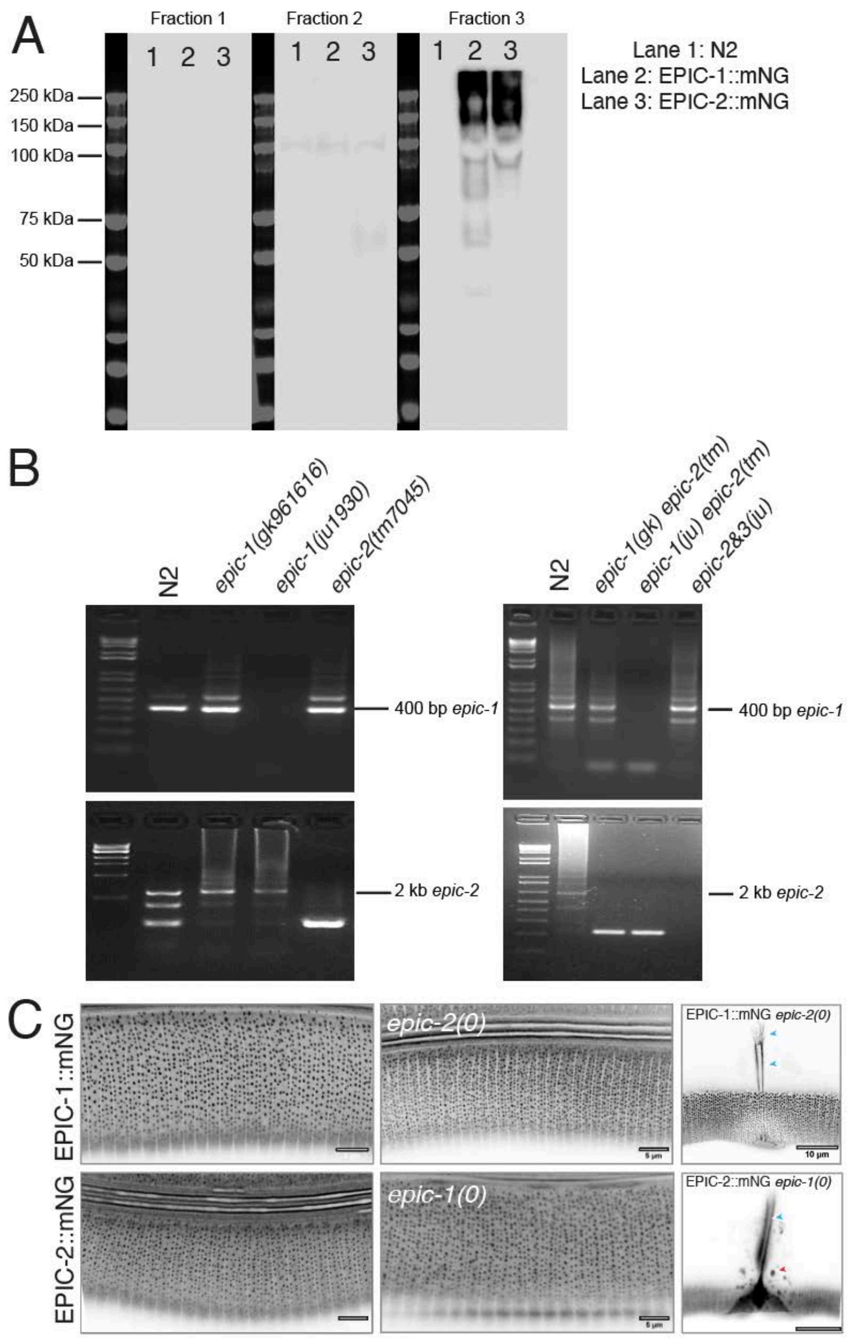

Fig. S2. EPIC biochemical, transcript and molecular epistasis analysis. A. Western blot of EPIC::mNG strains and N2 wild type controls. Mixed stage populations were prepared and fractions F1 (cytosolic), F2 (organelle), and F3 (cuticle) isolated (see methods). mNG fluorescence was verified during the cuticle isolation. Western blot probed with anti-mNG antibodies. EPIC::mNG proteins were not detected in F1 or F2 fractions and were detected in the

Development • Supplementary information

F3 cuticle fractions of knock-in strains. Faint bands could be seen at sizes that may correspond to monomeric proteins, however most signal was observed in high-molecular weight species or smears of ~150 kDa and larger. As EPIC proteins and their mNG fusion proteins are highly acidic (e.g. EPIC-1::mNG has a pI of 4.46) they may migrate slower than predicted in SDS-PAGE. The predicted molecular weights of untagged EPIC proteins after cleavage of the signal sequence are 33.7 kDa for EPIC-1 and 60.6 kDa for EPIC-2; isoelectric points (excluding signal sequences) are 3.99 (EPIC-1) and 4.15 (EPIC-2). B. RT-PCR analysis of *epic* transcripts in mutant strains. The major *epic-1* RT-PCR product is 400 bp, with weaker bands at larger or smaller sizes corresponding to other repeats. The major *epic-2* RT-PCR product is 2 kb, with smaller bands corresponding to internal repeats. C. EPIC-1::mNG localization appeared unchanged in *epic-2(tm7045)* mutant adults (L4+24h), showing strut and alae localization (left and middle panels), and localization to vulval cuticle (right panel, blue arrowheads). EPIC-2::mNG in appeared unchanged in *epic-1(ju1930)*, with localization in struts (left and middle panels), vulval cuticle (right panel, blue arrowhead), and vulval epithelium secretory vesicles (red arrowhead), similar to EPIC-2::mNG in WT (Fig. 3D). Maximum intensity projections of 5 confocal planes. Scales 5 µm (left and middle), 10 µm (right).

Development • Supplementary information

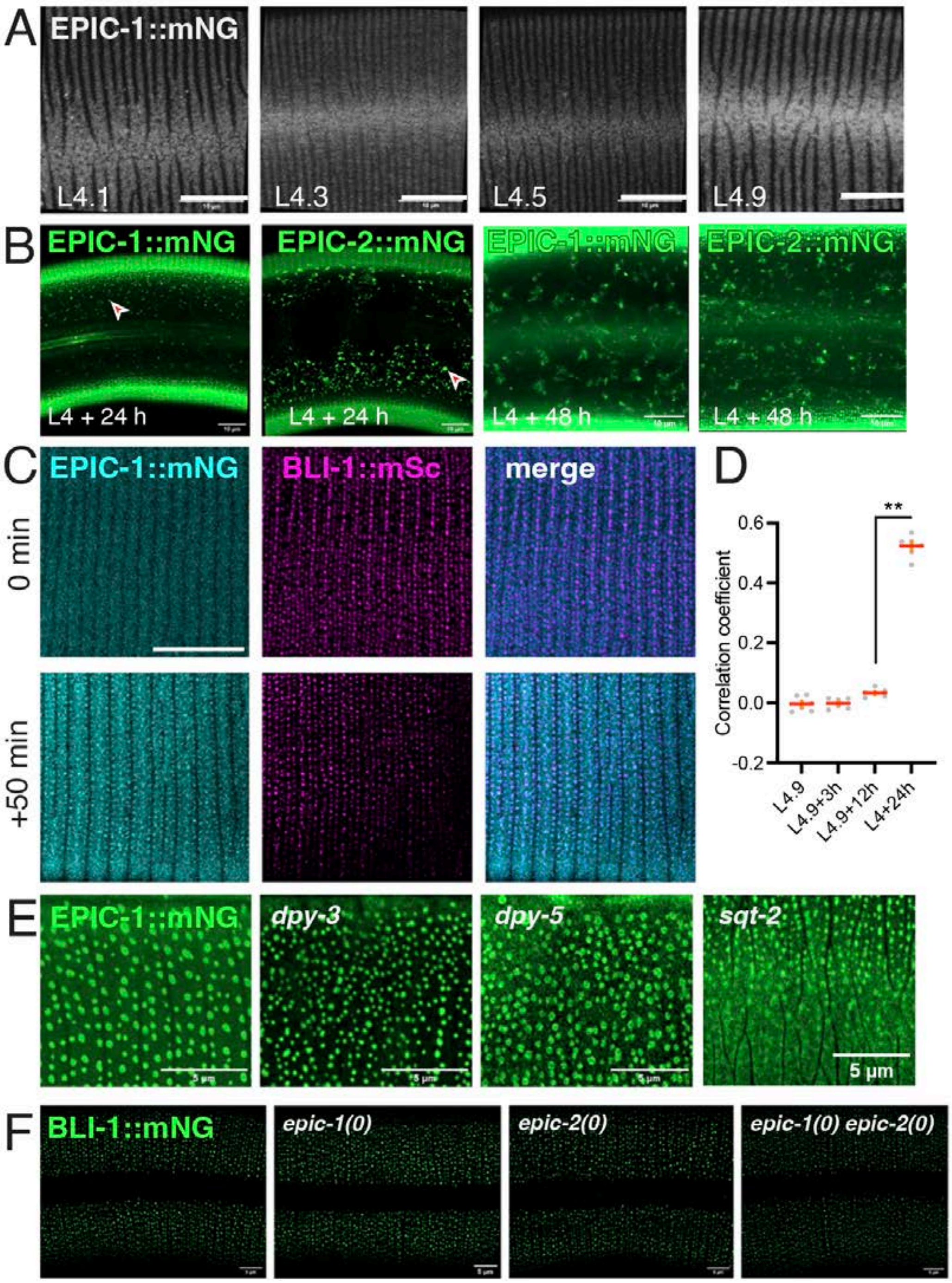

Fig. S3. Additional analysis of EPIC localization to adult struts. A. Localization of EPIC-1::mNG during L4 vulval morphology substages defined by Mok et al., 2015. Airyscan confocal, single focal planes; scale, 10 µm. B. Localization of EPIC-1::mNG and EPIC-2::mNG to putative secretory vesicles in the underlying epidermis in adults (L4 + 24 h and L4+ 48 h at

Development • Supplementary information

20°C); conventional confocal, maximum intensity projections of deep focal planes; Scales, 10 µm. C. Initial stages of EPIC-1 puncta formation in adult. EPIC-1::mNG (cyan) is non-punctate in early young adult (movie frame 2.9). Double label with BLI-1::mSc (magenta). EPIC-1::mNG puncta formation is visible ~50 min later (movie frame 3.47). Panels are 20 x 20 µm. Scale, 10 µm. D. Quantitation of BLI-1::mSc and EPIC-1::mNG colocalization in ROIs from L4.9-adult, Pearson's correlation coefficients. For the comparison of L4.9+12 h vs L4+24 h, P = 0.022 by Mann-Whitney test. E. EPIC-1::mNG strut patterning and localization in cuticle mutant adults *dpy-3(e182), dpy-5(e61)*, and *sqt-2(sc3)*. Airyscan confocal, single focal plane. Scales, 10 µm. F. BLI-1::mNG(*ju1789*) localization and pattern were normal in *epic-1(ju1930)*, *epic-2(tm7045)*, and *epic-1(ju1930) epic-2(tm7045)* backgrounds. Airyscan confocal, single planes. Scales, 10 µm.

Development • Supplementary information

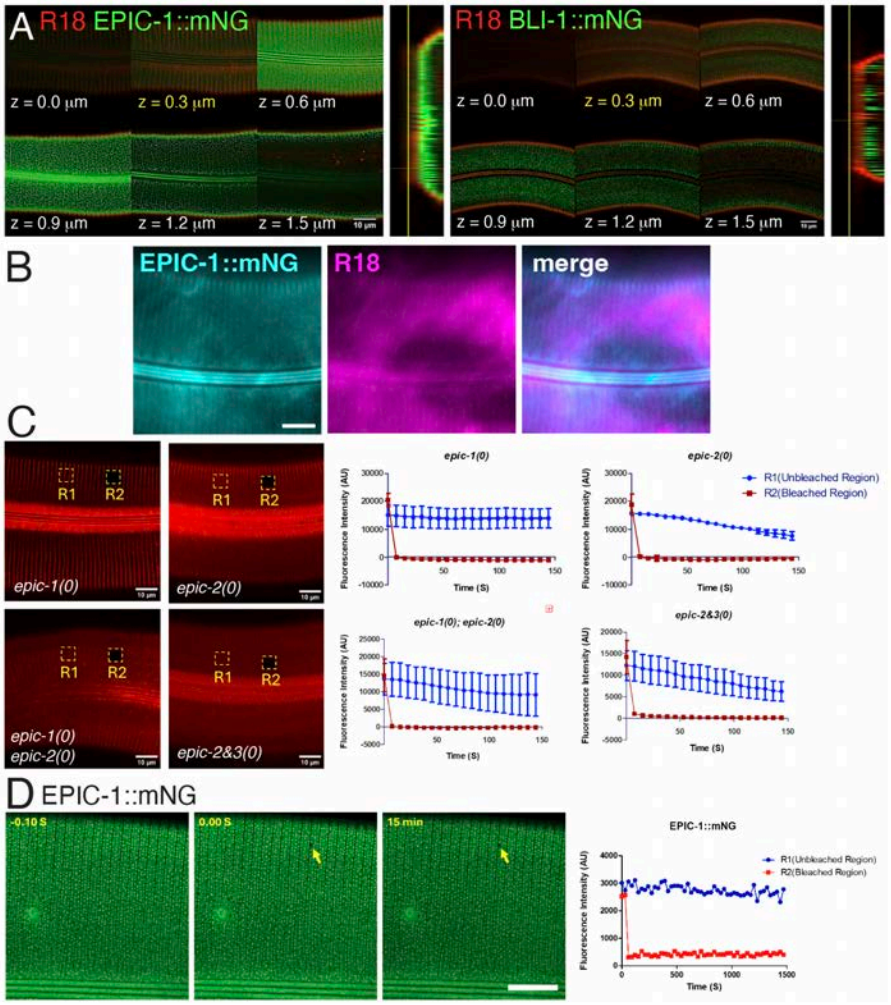

Fig. S4. Additional studies of cuticle lipophilic dye staining and EPIC mobility.
A. Montages and orthogonal views of R18 staining (red) in EPIC-1::mNG and BLI-1::mNG, Airyscan planes (6 x 0.3 µm thick). EPIC-1::mNG localized closer to the R18 staining than did BLI-1::mNG. Orthogonal sections (yellow line) at z = 0.3 µm. B. TIRF image of surface R18 and EPIC-1::mNG. Projection of 2 x 20 nm z slices centered in alae, xy scale 10 µm. C. FRAP of R18 lipid staining in *epic* single and double mutants. R18 signal did not recover in 2 min post bleaching in N2 or in *epic* mutants. Red lines, bleached areas (R2); blue lines, unbleached areas (R1); mean ± SEM of n = 3 FRAP experiments per genotype. D. FRAP of EPIC-1::mNG. Images are of pre-bleached, bleached (0 sec) and 15 min post bleached. Graph shows no recovery of fluorescence in bleached region and minimal imaging bleaching in unbleached region. Representative example of 3 FRAP experiments. Scale 10 µm.

Development • Supplementary information

Table S1. Molecular description of *epic* mutants and compound mutants, brood size and % lethality

Genotype	Genomic DNA	Predicted protein	Brood size (mean ± SEM)	Lethality
epic-1(ju1930)	1187 bp deletion	Deletes entire coding sequence	173 ± 46	0%
epic-1(ju1931)	885 bp deletion	Deletes repeats 1-4	292 ± 38	0.4%
epic-2(tm7045)	1776 bp deletion	Truncated after repeat 1	176 ± 48	0.1%
epic-2&3(ju2003)	3335 bp deletion	Deletes all of *epic-2* and *epic-3* coding sequence	210 ± 63	0.8%
epic-1(ju1930) epic-2(tm7045)			162 ± 49	0.4%
epic-1(ju1930) epic-2&3(ju2003)			170 ± 35	1.0%
epic-3(ju2045) and *epic-3(ju2053)*	1045 bp deletion	Deletes entire coding sequence	170 ± 35	0%

n = 10 broods counted per genotype. Lethality counts are based on >500 animals per genotype. Brood sizes and lethality (embryonic + larval) were measured at 20 °C; no additional lethal or abnormal phenotypes were observed at 15 °C or 25 °C.

Development • Supplementary information

Table S2. Strains and Genotypes

Strain	Genotype	Origin
CZ27940	*wSc::bli-1(ju1807) II*	J. Adams
CZ28797	*epic-2(tm7045) IV*	J. Adams
CZ28864	*epic-1(gk961616) IV*	J. Adams
CZ28898	*bli-2::halotag(syb4687) II; epic-1::mNG(syb4990) IV*	E. Jyo
CZ29069	*bli-1(ju1395) II; epic-1::mNG(syb4990) IV*	R. Iwazaki
CZ29311	*bli-1(ju1395) II; epic-2::mNG(syb5095) IV*	M. Pooranachithra
CZ29312	*bli-2(ju1380) II; epic-2::mNG(syb5095) IV*	M. Pooranachithra
CZ29317	*epic-1::mNG(syb4990) epic-2(tm7045) IV*	M. Pooranachithra
CZ29327	*epic-1(ju1930) IV*	M. Pooranachithra
CZ29557	*epic-1(ju1930) epic-2(tm7045) IV*	M. Pooranachithra
CZ29827	*epic-2::mNG(syb5095) IV*	M. Pooranachithra
CZ29944	*wSc::bli-1(ju1807) II; epic-1::mNG(syb4990) IV*	M. Pooranachithra
CZ29969	*epic-1::mNG(syb4990) IV; gmap-1(ulb13) X*	M. Pooranachithra
CZ30002	*epic-2&epic-3(ju2003) IV*	M. Pooranachithra
CZ30192	*epic-1(ju1930) epic-2&3(ju2003) IV*	M. Pooranachithra
CZ30198	*epic-1(ju1930) epic-2::mNG(syb5095) IV*	M. Pooranachithra
OQ192	*gmap-1(ulb13) X*	P. Laurent lab
PHX4990	*epic-1::mNG(syb4990) IV*	SunyBiotech
PHX5095	*epic-2::mNG(syb5095) IV*	SunyBiotech
PHX7470	*epic-3::mNG(syb7470) IV*	SunyBiotech
CZ30767	*bli-1(ju1395) II; epic-1(ju1930) IV*	M. Pooranachithra
CZ30749	*bli-1(e1431) II; epic-1(ju1930) IV*	M. Pooranachithra
CZ30750	*bli-2(e527ts) II; epic-1(ju1930) IV*	M. Pooranachithra
CZ30777	*mNG::bli-1(ju1789) II; epic-1(ju1930) IV*	M. Pooranachithra
CZ28863	*mNG::bli-1(ju1789) II; epic-2(tm7045) IV*	M. Pooranachithra
CZ30778	*mNG::bli-1(ju1789) II; epic-1(ju1930) IV epic-2(tm7045) IV*	M. Pooranachithra
CZ30771	*epic-3(ju2045) IV*	M. Pooranachithra
CZ30921	*epic-1(ju1930) epic-2&3(ju2003) IV; juEx8461[epic-1(+)]*	M. Pooranachithra
CZ30892	*bli-2(e527ts) II; epic-1::mNG(syb4990) IV*	M. Pooranachithra
CZ30903	*dpy-5::wSc(syb3326) I; epic-1::mNG(syb4990) IV*	A. Chisholm/K. Kang
CZ30904	*dpy-5::wSc(syb3326) I; epic-1::mNG(syb4990) IV; dpy-3(e182) X*	A. Chisholm/K. Kang
CZ30905	*dpy-5::wSc(syb3326) I; bli-1(ju1395) II epic-1::mNG(syb4990) IV*	A. Chisholm/K. Kang
CZ30799	*epic-1::mNG(syb4990) IV; dpy-3(e182) X*	C. Chen
CZ30793	*dpy-5(e61) I; epic-1::mNG(syb4990) IV*	C. Chen
CZ30560	*sqt-2(sc3) II; epic-1::mNG(syb4990) IV*	C. Chen

Development • Supplementary information

Table S3. Primers

Primer #	Target Gene	Primer Sequence	Allele covered
SD21029	*epic-1*	accatccaaccgtttataag	*gk961616/ju1930/ju1931*
SD21030		aaaacttgtggcagtgagat	*gk961616/ju1930/ju1931*
SD21031	*epic-2*	ATGGCCAGTAAGACCACTGT	*tm7045*
SD21032		CGGAGGAGCACAGGTCGTTG	*tm7045*
SD21033		CTCAACTGGAGCGTCGGCTG	*tm7045*
SD20527	*epic-2&3*	gtcggttggaaatggaagaagcagc	*ju2003*
SD20535		tgcgcgaaatattgtgcgtgagacacaaaggtcac	*ju2003*
SD20536		gcgagcgttagcaacgctatttatggttattaggc	*ju2003*
SD20471	*gmap-1*	ctgatatgttgtttgggataaagtttgatttcaag	*ulb13*
SD20472		tgtaaattttgttggttttacccggtcgtccactg	*ulb13*
SD20513		caactgattgggtcgccgggtagagcaatgtctcg	*ulb13*
SD20893	*epic-1 cDNA*	GAGAATAATCATTGTTGCTGGTCTTATCGC	*ju1930/gk961616*
SD20894		CGTAAGAGTTTTGTGCTTGTCTTTTAGATCTG	*ju1930/gk961616*
SD20890	*epic-2 cDNA*	GGTGTTTGTATTCCTCTCCATCGTTGCCAT	*tm7045*
SD20891		CTGGGGCTTGTTCTACTGGAGCGTCG	*tm7045*
SD20892		GGCTGGAGCAGCTGGAGCTGGGGCGGCAGA	*tm7045*
SD20895	*epic-3 cDNA*	GCTTGTTGGCTGTGTGACTGCTGCT	*ju2003*
SD20896		CAGCAGTCTTGGATCCTCCCGAGTT	*ju2003*
SD20484	*epic-3 mNG*	GATACCAAGTCCACCGTACC	*syb7470*
SD20999		ggctaactttgtcgatttgc	*syb7470*
SD21000		gacggaaaacctttatcattg	*syb7470*
SD21418	*epic-1 rescue*	CATCCAACCGTTTATAAGGACGACG	
SD21419		TTGCGCATTTATATAGGCCGCTTTG	
SD21254	*epic-3*	gcaagttgtggacggaaaacc	*ju2045*
SD20538		ggccacttactccaaatagcaaaac	

Development • Supplementary information

Table S4. Alleles and crRNAs for CRISPR

Gene	Deletion	Size	Flanking sequences
epic-1	*ju1930*	1187 bp	ATGAGAATAATCATTGTTGCTGGTCTTA
			CCCAGCTCAAGACGCTGGGTACAGAGCCTAA
epic-2&3	*ju2003*	3335 bp	cATGAAGGTGTTTGTATTCCTCTCCATCGTTGCCATC
			tcaagtatttaggtcatcaaacatttcgaaaaaacaaatattttcataagta
epic-3	*ju2045*	1029 bp	ggaatgcaacaagttacttatgaaaatatttgttttttcgaaatgtttga
			GGATCCAAGACTGCTGTCGAATCCAGTGGTTACTGAtttctg attcaatt
CRISPR crRNA sequences			
	Name	Gene Target	Sequence
	crPM1_epic-2_crRNA1	*epic-2*	GCTGCTCCAGATGTTGAGTG
	crPM2_epic-3_crRNA1	*epic-3*	GCTGTGACTGTGGAAAACTC
	crPM3_epic-1_crRNA1	*epic-1*	TGTTGCTGGTCTTATCGCCT
	crPM4_epic-1_crRNA2	*epic-1*	TCAGGAAGAGACAGCTCCAG
	crPM5_epic-2_crRNA2	*epic-2*	ACCGTAGCGAGAAGAAGCGA
	crPM6_epic-3_crRNA2	*epic-3*	TGACCTAAATACTTGAGAAC

Development • Supplementary information
